# *Lamellodiscus euzeti* n. sp. (Monogenea: Diplectanidae), a parasite from *Dentex canariensis* and *D. gibbosus* (Teleostei: Sparidae) in the Atlantic Ocean and Mediterranean Sea

**DOI:** 10.1051/parasite/2011182145

**Published:** 2011-05-15

**Authors:** A. Diamanka, L. Boudaya, B.S. Toguebaye, A. Pariselle

**Affiliations:** 1 Laboratory of Parasitology and Laboratory of Aquatic Animals Pathologies-Khaled Bin Sultan Living Oceans Foundation, Department of Animal Biology, Faculty of Sciences and Technologies, University Cheikh Anta Diop Dakar Senegal; 2 UR 203, UMR 5554, IRD/ISE-M, Laboratoire d’Écologie et de Systématique, Campus de Bel Air BP 1386 Dakar Sénégal; 3 Laboratoire de Biodiversité et Écosystèmes Aquatiques, Faculté des Sciences de Sfax, Université de Sfax BP 1171 3000 Sfax Tunisie

**Keywords:** Monogenea, Diplectanidae, *Lamellodiscus euzeti*, *Dentex canariensis*, *Dentex gibbosus*, Sparidae, Ivory Coast, Senegal, Tunisia, Monogenea, Diplectanidae, *Lamellodiscus euzeti*, *Dentex canariensis*, *Dentex gibbosus*, Sparidae, Côte d’Ivoire, Sénégal, Tunisie

## Abstract

*Lamellodiscus euzeti* n. sp. (Monogenea: Diplectanidae) is described from the gills of two sparid fishes, *Dentex canariensis* (Steindachner) off Senegal and Ivory Coast and *D. gibbosus* (Rafinesque) off Senegal and Tunisia. The new species belongs to the “ignoratus” group, characterized by a lamellodisc with complete lamellae, a “lyre” shaped male copulatory organ type, and the “ignoratus” *sensu stricto* subgroup, characterized by a haptor with simple lateral dorsal bars. *Lamellodiscus euzeti* n. sp can be distinguished from all the congeneric species of the “ignoratus” subgroup by the presence of a prominent protuberance at the base of the curved part of the simple piece of the male copulatory organ (MCO), a large bulb at the base of the bifurcated piece of the MCO and the presence of 5-6 spines in the distal portion of the axial branch of the bifurcated piece of the MCO. Specificity and biogeography of *Lamellodiscus* species from sparid fishes are discussed.

## Abstract (fr)

Les auteurs décrivent *Lamellodiscus euzeti* n. sp. (Monogenea : Diplectanidae) sur les branchies de deux Sparidae, *Dentex canariensis* (Steindachner) échantillonné au large du Sénégal et de la Côte d’Ivoire et *D. gibbosus* (Rafinesque) échantillonné au large du Sénégal et de la Tunisie. Cette nouvelle espèce appartient au groupe “ignoratus”, caractérisé par un hapteur avec des lamellodisques à lamelles complètes, un organe copulateur mâle (OCM) en forme de “lyre”, et au sous-groupe “ignoratus” *sensu stricto*, caractérisé par un hapteur avec des barres latérales dorsales simples. *Lamellodiscus euzeti* n. sp se distingue de tous ses congénères du sous-groupe “ignoratus” par la présence d’une protubérance proéminente à la base de la partie courbe de la pièce simple de l’OCM, d’un large bulbe à la base de la pièce fourchue de l’OCM, et de cinq à six épines sur la partie distale de la branche axiale de la pièce fourchue de l’OCM. La spécificité et la biogéographie des espèces de *Lamellodiscus* sont discutées.

## Introduction

*Lamellodiscus* Johnston & Tiegs, 1922 (Monogenea: Diplectanidae) is currently composed of 56 described species (see Euzet & Oliver, 1967; Kritsky *et al.*, 2000; Amine & Euzet, 2005; Amine *et al.*, 2006, 2007; Neifar, 2008; Boudaya *et al.*, 2009; Justine & Briand, 2010; Diamanka *et al.*, 2011) that have been mainly studied in sparids. During our survey on the diversity and specificity of diplectanid (Monogenea) parasites of Atlantic and Mediterranean Sparidae (see Diamanka *et al.*, 2011); we have collected from the gills of *Dentex canariensis* (Steindachner, 1881) from the coast of Senegal near Dakar, a *Lamellodiscus* belonging to the “ignoratus” *sensu stricto* subgroup. This species was also found on the gills of *Dentex gibbosus* (Rafinesque, 1810) from the same location and from Sfax fish market (Tunisia). According to the morphology of its male copulatory organ (MCO), this parasite differs from all currently known *Lamellodiscus* species and so is a new species. It description is given below, and specificity of *Lamellodiscus* species is discussed.

## Materials and Methods

**Fig. 1.**
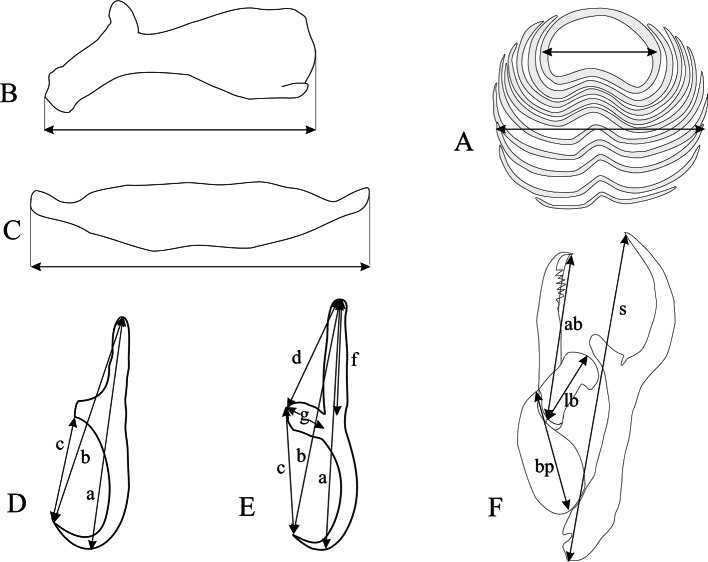
Measurements of sclerotised organs. A. lamellodisc, B. dorsal bar, C. ventral bar, D. dorsal anchor: a. total length, b. shaft-point distance, c. guard-point distance, E. ventral anchor: a. total length, b. shaft-point distance, c. guard-point distance, d. shaft-guard distance, f. shaft length, g. guard length, F. MCO: s. simple piece total length, paired piece: bp. basal part, ab. axial branch, lb. lateral branch.

Twenty seven (27) specimens of *Dentex canariensis* and 48 of *Dentex gibbosus* were collected from Soumbedioune and Ouakam fish markets, near Dakar in Senegal, and four *D. gibbosus* from Sfax fish market, in Tunisia. They were identified using Blache *et al.* (1970) and Bellemans *et al.* (1988) in Senegal and Fischer *et al.* (1987) in Tunisia; distributions areas are estimated after FishBase (2009). Fish were dissected immediately or frozen until examination. Gills arches were removed and placed in separate Petri dishes containing seawater and examined for parasites under incident light using stereomicroscope. Monogeneans were detached from the gills with strong water current and transferred to a dish containing filtered seawater. Some parasites were partially compressed beneath slide and coverslip and examined using an optical microscope. Others parasites were transferred and mounted onto a slide in a drop of ammonium picrate-glycerol fluid (Malmberg, 1957). The preparation was then covered with a round coverslip and sealed with Glyceel (Bates, 1997). Drawings were made using a Leitz microscope equipped with a drawing tube and they were scanned and redrawn with Corel Draw Software. Measurements, made on Malmberg’s fixed specimens and taken using a DM2500 Leica microscope, a DFC320 Leica digital camera and the Leica Application Software v 3.0, are given in micrometers as the mean ± standard deviation followed in parentheses by the range and (n) the number of observations. Terms and measures of the sclerotised structures are those proposed by Neifar *et al.* (2004) and presented in Fig. 1. Types were deposited in the collections of the Muséum National d’Histoire Naturelle (Paris) (MNHN) and of the British Natural History Museum (London) (BNHM).

## Results

**Fig. 2.**
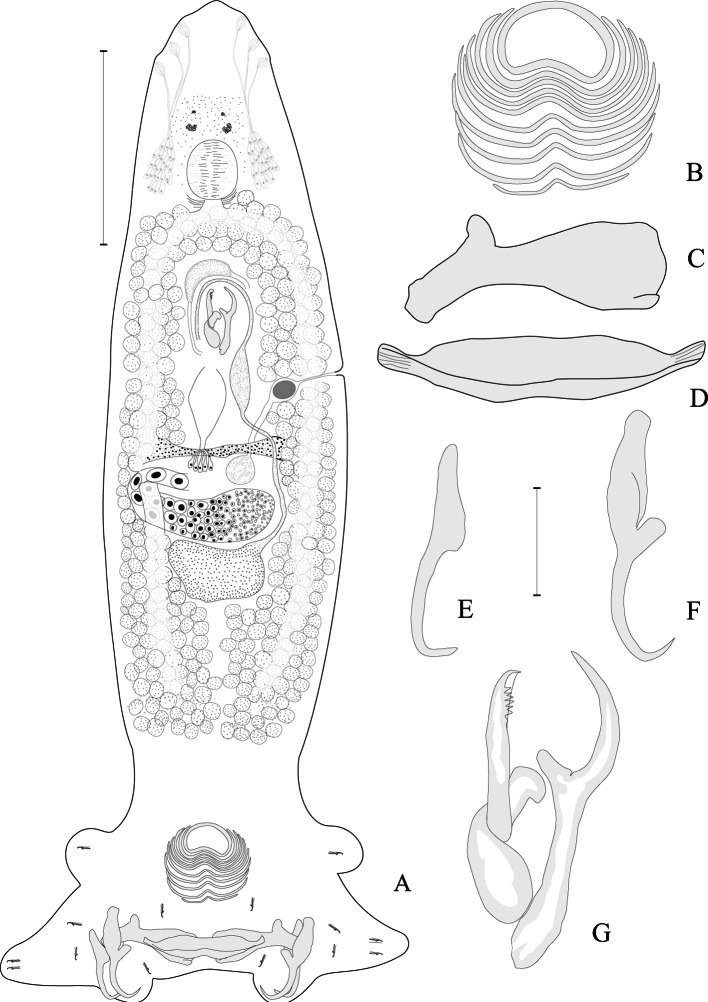
*Lamellodiscus euzeti* n. sp. from the gills of *Dentex canariensis* off Dakar, Senegal. A. animal *in toto* ventral view, composite drawing of three living and fixed specimens, B. lamellodisc (elegans type), C. dorsal bar, D. ventral bar, E. dorsal anchor, F. ventral anchor, G. male copulatory organ. Scale-bar A = 250 μm; B, C, D, E, F & G = 25 μm.

*Lamellodiscus euzeti* n. sp. (Fig. 2)

Type host: *Dentex canariensis* (Steindachner, 1881) (Sparidae).

Other host: *Dentex gibbosus* (Rafinesque, 1810) (Sparidae).

Site: gills, between secondary gill lamellae.

Type locality: off Dakar, Senegal.

Other records: Sfax fish market, Tunisia on *D. gibbosus* and Abidjan fish market, Ivory Coast on type host (Euzet, pers. comm.).

Material studied: 31 specimens mounted in ammonium picrate-glycerol and five live specimens.

Global prevalence: 70%; 55 infected of 79 fish examined.

Prevalence on *D. canariensis* from off Senegal: 70%; 19 infected of 27 fish examined.

Prevalence on *D. gibbosus* from off Senegal: 67%; 32 infected of 48 fish examined.

Prevalence on *D. gibbosus* from Tunisia: 100%; four infected of four fish examined.

Etymology: the name “euzeti” is given in honour of Louis Euzet professor emeritus from the University of Montpellier (France), who had already collected and began the study of this species in Ivory Coast in 1972.

Type material: holotype (MNHN n° HEL 188 Th 207) and paratypes (MNHN n° HEL 189 Th 208, n° HEL 190 Th 209, BMNH n° NHMUK 2011.2.17.1-3).

### • Description

Diplectanidae, Lamellodiscinae. Adults 1142 ± 167 (802-1622, n = 31) long including haptor; maximum width 211 ± 33 (135-280, n = 31) at level of ovary. Haptor 251 ± 34 (134-314, n = 31) wide, with two lateral lobes on each side (Fig. 2A). Dorsal and ventral lamellodiscs, 52 ± 3.5 (46-62, n = 62) in diameter, with ten concentric rows of lamellae; anterior forming complete ring 29 ± 2.3 (24-36, n = 62) in diameter. Dorsal anchors with incipient guards, a: 48 ± 1.5 (44-51, n = 62); b: 45 ± 1.4 (42-48, n = 62); c: 21 ± 1.5 (18-28, n = 62). Two lateral dorsal bars with spatulate inner ends and a prominent protuberances at outer third of each bars, 64 ± 1.9 (60-68, n = 62) long and 23 ± 0.9 (20-24, n = 62) wide. Ventral anchors with well marked handles, wide guards, bent shafts and an acute points, a: 55 ± 2.7 (49-61, n = 62); b: 51 ± 2.6 (47-56, n = 62); c: 28 ± 2.1 (21-33, n = 62); d: 25 ± 1.8 (21-29, n = 62); f: 31 ± 1.3 (28-34, n = 62); g: 12 ± 1.0 (9-14, n = 62). Ventral median bar 77 ± 2.0 (74-82, n = 31) long and 20 ± 1.9 (16-23, n = 31) wide, with curved ends. Fourteen similar uncinuli (seven pairs) 12 ± 0.7 (11-14, n = 90) long, with diplectanid disposition (Fig. 2A).

Cephalic glands on each side of the pharynx with ducts leading to three lateral head organs. Four granular ocellar pits forming two pairs, posterior pairs greater than the anterior pairs, and larval ocellar granules sometimes dispersed all around the pharynx. Mouth anterior, subterminal, opening ventrally. Pharynx spherical, 86 (78-95, n = 16) in diameter. Oesophagus short. Simple lateral intestinal caeca not joined posteriorly. Subspherical testis, intercaecal in posterior half of the body. Vas deferens emerging from antero-sinistral side of testis, not encircling left intestinal caecum, enlarging to form seminal vesicle. Vas deferens passing on right side, anterior to MCO. Prostatic reservoir pyriform, anterior to MCO. MCO of “lyre” morphology, with two sclerotised, articulated pieces (Fig. 2G). Simple piece ending in a strong hook with a prominent protuberance at the base of the distal third, s: 75 ± 1.9 (71-79, n = 31) long. Bifurcated piece with a large basal part, bp: 25 ± 2.6 (21-34, n = 31) long, and two unequal crossed branch; the lateral one, shorter, extends intersecting the axial one at proximal extremity, lb: 20 ± 1.5 (15-23, n = 31); the axial one with 5-6 spines near its slightly curved distal extremity, ab: 41 ± 2.4 (34-46, n = 31). Ovary median, subequatorial, anterior to testis, looping right intestinal caecum. Mehlis’s glands and ootype present. Vaginal aperture sinistral with funnel-shaped opening, enlarged on vaginal chamber often filled with slightly sclerotised globular mass. Vaginal chamber connected with narrow duct to globular seminal receptacle, anterior to ovary. Vitelline follicles lateral, coextensive with intestinal caeca, contiguous anteriorly and posteriorly *in vivo*, left and right follicles overlapping in mounted specimens (see Fig. 2A). Eggs tetrahedral *in utero* (not seen outside the uterus).

### • Remarks

From the morphology of the squamodiscs, dorsal lateral bars and MCO, this new species belongs to the “ignoratus” group (*sensu* Oliver, 1987), characterized by lamellodiscs with complete lamellae and a “lyre” type male copulatory organ, and to the “ignoratus” *sensu stricto* subgroup, characterized by a haptor with simple lateral dorsal bar as proposed by Amine & Euzet (2005). This subgroup today includes 16 species: *L. pagrosomi* Murray, 1931; *L. ignoratus* Palombi, 1949; *L. fraternus* Bychowsky, 1957; *L. erythrini* Euzet & Oliver, 1967; *L. knoepffleri* Oliver, 1969; *L. acanthopagri* Roubal, 1981; *L. sarculus* Neifar, Euzet & Oliver, 2004; *L. sigillatus* Neifar, Euzet & Oliver, 2004; *L. rastellus* Neifar, Euzet & Oliver, 2004; *L. falcus* Amine, Euzet & Kechemir-Issad, 2006; *L. neifari* Amine, Euzet & Kechemir-Issad, 2006; *L. confusus* Amine, Euzet & Kechemir-Issad, 2007; *L. crampus* Neifar, 2008; *L. vicinus* Diamanka, Neifar, Pariselle & Euzet, 2011, *L. toguebayei* Diamanka, Neifar, Pariselle & Euzet, 2011 and *L. triacies* Diamanka, Neifar, Pariselle & Euzet, 2011. *Lamellodiscus euzeti* n. sp. can be easily distinguished from all these species, except *L. sarculus*, by the shape and size of the sclerotised pieces of the haptor and MCO. *L. euzeti* differs from *L. sarculus* by the length of the lateral branch (shorter in *L. sarculus*), the presence of 5-6 spines on the axial branch (*vs* smooth in *L. sarculus*), the bulbous shape of the basal part of the bifurcated piece of the MCO, and by the length of the protuberance at the base of the distal third of the simple piece (larger in *L. euzeti*).

## Discussion

The globular mass we observed in the vaginal chamber of *L. euzeti* is regularly present in different species of *Lamellodiscus* belonging to the “ignoratus” subgroup (Kritsky *et al.*, 2000; Neifar *et al.*, 2004; Amine *et al.*, 2006; Diamanka *et al.*, 2011). This globular mass seems to be characteristic to these species with a “lyre” type MCO, except for *L. triacies* in which it is smaller and resembles a spermatophore (Diamanka *et al.*, 2011). *L. euzeti* is described from two congeneric host species, *Dentex canariensis* and *Dentex gibbosus*, which raises the problem of its specificity, usually described as very strict in *Lamellodiscus* (Amine *et al.*, 2006, 2007; Amine & Euzet, 2005; Boudaya *et al.*, 2009; Euzet & Oliver, 1967; Neifar *et al.*, 2004; Oliver, 1987). In this case (other hypothesis has been developed by Desdevises *et al.*, 2002) the presence of this species on these two sparid fishes can be explained by their phylogenetic proximity, which may favour lateral transfers or exchanges of genes between populations of parasites (Combes, 1995; Pariselle, 2003). That seems to be the case since *D. canariensis* and *D. gibbosus* are very close phylogenetically (Chiba *et al.*, 2009) and are present in sympatry over much of their distribution area, from Western Sahara to Angola (and recently reported in Spain); and from Portugal to Angola, also in the Mediterranean Sea and around the Canary, Sao Tome and Principe islands respectively. The presence of this species in West Africa and Tunisia raises to seven the number of *Lamellodiscus* species described in the Atlantic Ocean and the Mediterranean Sea. Justine (1985) reported in *Diplodus sargus cadenati* de la Paz, Bauchot & Daget, 1974, three species of *Lamellodiscus* (*L. elegans* Bychowsky, 1957; *L. ergensi* Euzet & Oliver, 1966 and *L. ignoratus*) already known in the Mediterranean Sea on *D. sargus* (Linnaeus, 1758); Neifar *et al.*, 2004 described *Lamellodiscus sarculus* and *L. sigillatus* from *Pagrus caeruleostictus* and *L. rastellus* from *P. auriga*, suggesting a common history of Sparidae and their parasites in these distant areas, despite their ecological differences. Research is ongoing on other sparid species (and their parasites) to strengthen future comparisons between Atlantic and Mediterranean faunas.
